# Defining reference values of arterioles in healthy individuals for studies with adaptive optics imaging

**DOI:** 10.3389/fopht.2024.1348900

**Published:** 2024-03-22

**Authors:** Friederike C. Kortuem, David A. Merle, Milda Reith, Laura Kuehlewein, Ronja Jung, Saskia Holocher, Krunoslav Stingl, Katarina Stingl, Melanie Kempf

**Affiliations:** ^1^ University Eye Hospital, Center for Ophthalmology, University of Tübingen, Tübingen, Germany; ^2^ Institute for Ophthalmic Research, Center for Ophthalmology, University of Tübingen, Tübingen, Germany; ^3^ Department of Ophthalmology, Medical University of Graz, Graz, Austria; ^4^ Center for Rare Eye Diseases, University of Tübingen, Tübingen, Germany

**Keywords:** retinal vessels, wall-to-lumen ratio (WLR), wall cross-sectional area (WCSA), lumen diameter (LD), hypertension, adaptive optics imaging, ageing

## Abstract

**Purpose:**

To investigate age-dependent wall to lumen ratio (WLR) reference values for healthy individuals in adaptive optics imaging (AO). WLR serves as an objective, dimensionless parameter for the evaluation of structural changes in vessels caused by conditions like arterial hypertension, diabetes or vascular stenosis.

**Methods:**

50 right eyes of healthy individuals were examined by adaptive optics imaging. The central big arterioles and smaller arterial branches at least one disc diameter away from the optic disc, approximately above or below the macula were measured by the manufacturer’s software. The wall-lumen-ratio (WLR), the wall cross-sectional area (WCSA) and lumen diameter (LD) were assessed. Subsequent data analysis was performed with a focus on variables including age, gender and blood pressure.

**Results:**

Normative values for WLR, WCSA and LD in 5 different age groups could be established. However, no significant differences between the age groups were found. Intra-subject comparisons revealed significantly higher WLRs on peripheral branches when compared to central arterioles. WLR showed in this normotensive cohort no relevant correlation with the systolic, diastolic and mean blood pressure. Gender and intraocular pressure had no influence on the vascular parameters.

**Conclusion:**

AO is capable of examining vascular alterations in arterioles at an almost microscopic level. Age did not seem to alter WLR, normotensive blood pressure parameters showed also no significant impact. AO-based vessel analysis may provide clinically useful biomarkers for cardiovascular health and should be tested in future studies.

## 1 Introduction

Retinal vascular changes are common sequelae of systemic vascular diseases like arterial hypertension or diabetes but are also a hallmark of retinal degenerative or inflammatory diseases.

In comparison to the choroidal vessels, the retinal vasculature possesses the ability of autoregulation. There is evidence that the retina and the optic disc keep a constant blood perfusion in the vessels independent of changes in systemic perfusion or intraocular pressure (1). Riva et al. showcased that in healthy individuals, there is an adaptive response to an artificially elevated intraocular pressure of 29 mmHg, manifested as a decrease in perfusion pressure (2). Whereas an increase of the retinal vascular resistance to an increased perfusion pressure could be observed that was independent of any metabolic or hormonal parameters (1). Studies on cerebral hyperemia could also demonstrate that not the capillaries but the arterioles regulate the blood flow (3, 4). In contrast, the choroid is subject to neurogenic regulation (1). With aging this autoregulative ability seems to decrease, as the musculature in the vascular media undergoes gradual atrophy (5).

**Figure 1:**
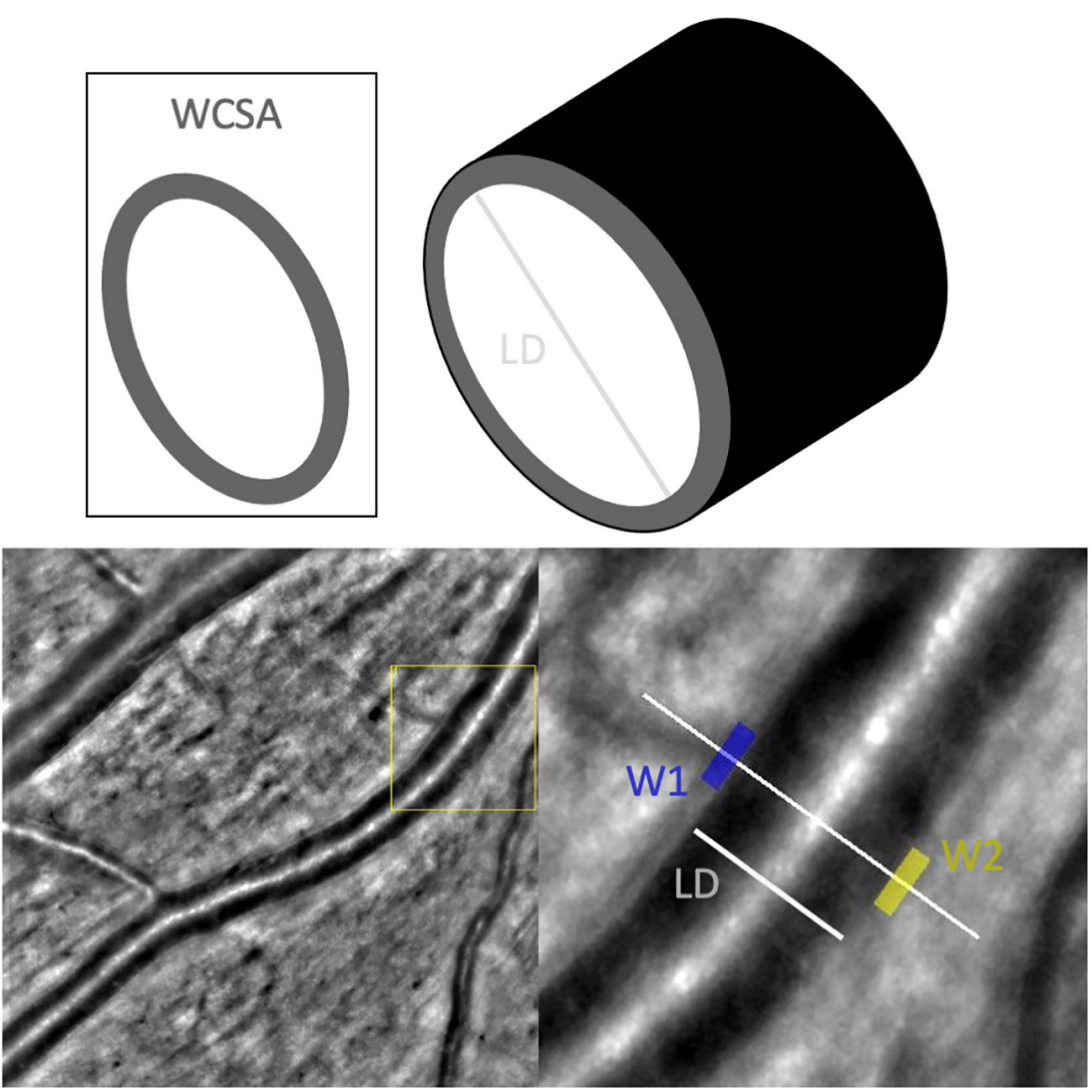
Measurements taken of the vasculature by AO-Detect software. WCSA, wall cross-sectional [μm^2^]; LD, lumen diameter; W1, wall 1 [μm]; W2, wall2 [μm].

Adaptive optics imaging (AO) allows non-invasive *in vivo* imaging at high spatial, near microscopic level resolution, making photoreceptors and details of vascular architecture visible. Therefore, AO offers the possibility to detect subtle changes in an early-stage diseases and may facilitates new diagnostic and therapeutic approaches. For the assessment of vessels with AO certain parameters and norms for the description were defined by Bakker et al. (6). Structural changes in the vasculature can be objectively assessed using the wall-to-lumen ratio (WLR) as a nondimensional unit of level of stenosis or *via* other parameters such as the lumen diameter (LD) and wall cross-sectional area (WCSA) (**Figure 1**).

Until today, AO-based studies on the vascular architecture mainly focused on vascular diseases such as diabetic retinopathy (DR) (7), hypertension (8–10) and retinal vasculitis (11).

Zalenska-Zmijewska et al. could demonstrate that the cone density and regularity decreased with increasing severity of DR. Here, the arterial walls were significantly thicker in the DR group. The wall lumen ration (WLR) and wall cross-sectional area WCSA differed significantly between DR patients and healthy subjects (7).

Koch et al. analyzed changes in small retinal vessels in treatment-naïve individuals. Subjects partly had an elevated, but untreated hypertension. They found that WLR positively correlated with the mean blood pressure and age (9). The reference area for the measurements was defined as a superotemporal arteriole of a right eye with a diameter of at least 50 mm located one disc diameter away from the optic disc.

Other studies on hypertension used scanning laser Doppler flowmetry (SLDF) (12–14) and suggested inner diameter, parietal thickness and outer diameter as relevant parameters.

Another AO-based study on vascular changes by our group focused on postinterventional vascular changes in patients with *RPE65-*associated inherited retinal degeneration treated with voretigene neparvovec (15). Here, only small vessels were reliably measurable due to severe alterations in the degenerated retina. However, depending on the phenotype in other retinal degenerative diseases, measurements at central arterioles may provide valuable information.

Even though the mentioned studies were able to find promising vascular changes, we obligatorily need to understand normative values and their potential confounding factors, like for example age, to enable clinically meaningful measurements and their interpretation. Therefore, this study aimed to establish reference values for the WLR, LD and WCSA in healthy individuals with normotensive blood pressure and assess variables that could influence those.

**Figure 2:**
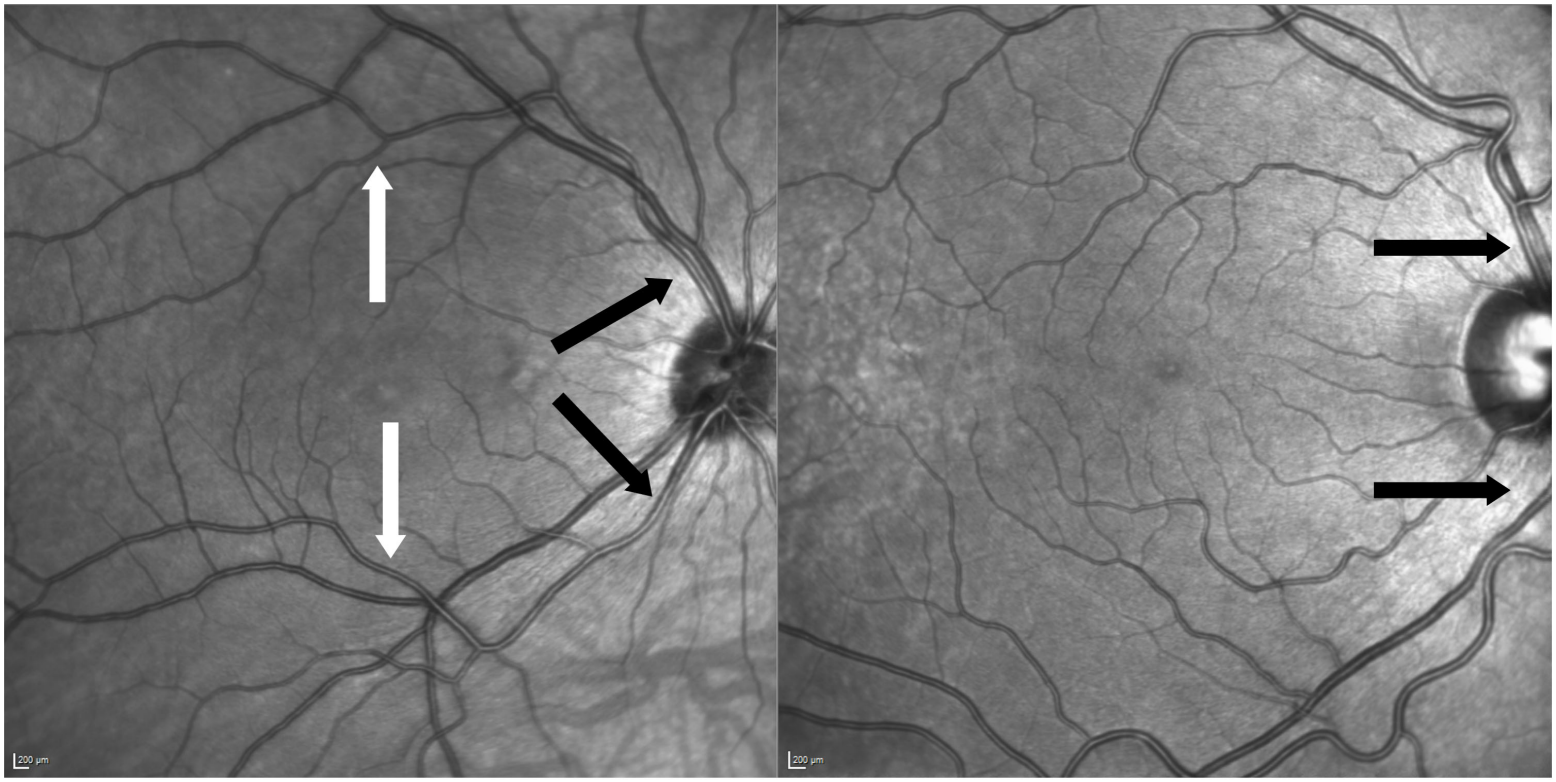
Two examples of anatomic variants of vessel formation. Left picture of central arteriole and branch measured of the same patient (black arrows indicate central locations and white arrows above the macula on arterial branches). Right picture shows non-measurable anatomic variant due to crossing venules and nasalization.

For this study vessels at two locations were evaluated: Firstly, central arterioles within one optic disc diameter above or below the optic disc. Secondly, branches of the central arteriole that were situated beyond one optic disc diameter away, extending towards the vascular arcades and more centrally near the macular region. Through this approach, it is possible to establish and compare two reference points within the same individual, each corresponding to vessels of a specific diameter (see **Figure 2**).

## 2 Materials and methods

50 eye-healthy individuals were examined by high-resolution retinal imaging using the adaptive optics flood illumination rtx1™ system (Imagine Eyes, Orsay, France).

Enrolled subjects had no vascular relevant comorbidities such as diabetes. In addition to age, we assessed other potentially relevant parameters, including blood pressure, gender and intraocular pressure (IOP).

The subjects were between 20 to 69 years old and equally divided into five decadic age groups (20-29; 30-39; 40-49; 50-59; 60-69 years). Minimum best corrected visual acuity (BCVA) was set to 20/25. Persons with known retinal or ocular inflammatory diseases, amblyopia, myopia magna or relevant opacities were excluded.

Normal blood pressure was defined as systolic less than 140mmHg (systolic blood pressure SBP), diastolic less than 90 mmHg (diastolic blood pressure DBP). The mean blood pressure (MBP) was derived by the formula:


$$MBP=\;DBP\;+\;\frac{1}{3}\left(SBP-(DBP\right)$$

Individuals were only included if the blood pressure was within this range. All study participants had no blood pressure lowering medication.

The intraocular pressure (10-21 mmHg) was normal without any reported medication. Pressure was measured by Goldmann applanation tonometry. The measurements were only taken once and, in each case, the right eye was defined as the study eye. Pupil dilatation was necessary in older individuals (mainly above 50 years) as the pupil did not dilate sufficiently for a satisfactory resolution. A b-scan using the IOL Master 700 (Carl Zeiss Meditec, Jena, Germany) was performed to measure the axial length of the bulbus as the axial length is taken into account by the rtx1 system during image capture and parameter calculation.

Measurements were taken at two different locations: Firstly, central arterioles of the temporal arcade near the optic disc were assessed. Only arterioles that directly left the optic disc and emerging from the central retinal artery were measured. In clinical practice these arterioles are often referred to ‘arteries’ of the upper and lower vascular arcade. They were not further from the optic disc than one disc diameter. In the rtx software coordinates were around 10-13N° and +/-6-10° depending on measured above or below the optic disc.

Secondly, branches of the arterioles further away than one optic disc diameter were measured. Branches right above or below the macula were preferred. In the rtx software the points measured above or below the macula translated into coordinates of 1.5T° up to 1.5N° and +/-6-7°.

Only healthy individuals who had this measurable configuration were included in this study (see **Figure 2**). Five measurements were taken at both locations, the chosen central and smaller arterial branch. The retinal arterioles were measured by using AOdetect Artery semiautomated software (Imagine Eyes, Orsay, France Version 3.0). All measurements were taken and adjusted by the same examinator.

The used outcome parameters were:

○ the lumen diameter (LD)
○ the wall lumen ratio (WLR):

calculated automatically by the software as the ratio of


$$\frac{\left(W1+W2\right)}{LD}$$

with W1 and W2 being the thickness values of the transected vessel walls

○ the wall cross-sectional area (WCSA).

WCSA as the area encompassed by vessel circumference-lumen area:


$$\frac{\pi }{4}\left(TD^{2}-LD^{2}\right)$$

With total diameter (TD) = LD+W1+W2 (see **Figure 1**)

Resulting in:


$$\frac{\pi }{4}\left({\left(LD+W1+W2\right)}^{2}-LD^{2}\right)$$

Measurements were given in micrometers. For all subjects, the arithmetic mean of WLR, LD and WCSA was derived from five individual measurements, both, for the central arteriole and at the peripheral branches.

For all subjects, the blood pressure and the intraocular pressure were documented. The AO-derived vascular parameters were then correlated to IOP, gender and age. Statistical analysis was performed using SPSS (Version 27.0 SPSS Inc. Chicago, IL). For testing intra-observer consistency, three repeated measures with the software were performed in half of the patients and Cronbach’s alpha assessed.

The threshold for statistical significance was set to a p-value of less than 0.05. Normal distribution was assessed with the Kolmogorov-Smirnov-Test. Correlation analysis was performed using Pearson’s correlation coefficient for normally distributed data. T-test or ANOVA were calculated to compare groups. Multiple testing correction was conducted using Bonferroni correction. This study was approved by the ethic commission of the University Hospital Tübingen, Germany (660/2017BO1) and was following the principles of the Declaration of Helsinki. Written consent was obtained from all participants.

## 3 Results

Cronbach’s alpha was over 0.7 for WLR, WCSR and LD. The examined subjects had an average age of 44.80 (±14.015) years. The majority were female [n_female_=33 (66%); n_male_= 17 (34%)].

The average axial length was 23.72 (±1.06) mm. The refraction ranged from +2,00 dpt to -4,00 dpt spheric. Most included individuals (n=24) were emmetropic with an axial length under 24 mm. 17 were myopic, but only four had a higher myopia than -3,0 dpt. The rest was hyperopic (n=9).

The mean IOP was 13.60 (±2.38) (see **Table 1**). The measured IOP was normally distributed (Kolmogorov-Smirnov *p*=0.200). The parameters for blood pressure were all normally distributed (Kolmogorov-Smirnov *p*_SPB_=0.2; *p*_DBP_=0.055; *p*_MBP_=0.2). For all subjects, measurements were taken at central arterioles and at smaller branches.

**Table 1:** Descriptive statistics of the independent variables; n=50, BP, blood pressure.

	min	max	mean	standard error	standard deviation
**age[years]**	24	69	44.80	1.98	14.02
**IOP [mmHg]**	9	8	13.60	0.33	2.38
**systolic BP [mmHg]**	106	136	124.32	1.12	7.90
**diastolic BP [mmHg]**	54	90	80.84	1.12	7.91
**mean BP [mmHg]**	73	105	95.33	0.99	6.98
**axial length [mm]**	21.31	25.85	23.72	0.15	1.06

In funduscopic examination and in AO imaging no hypertensive vascular changes such as diffuse narrowing of arterioles and focal lesions such as focal arteriolar narrowing (FAN) and arteriovenous nicking (AVN) were evident.

### 3.1 WLR

**Figure 3:**
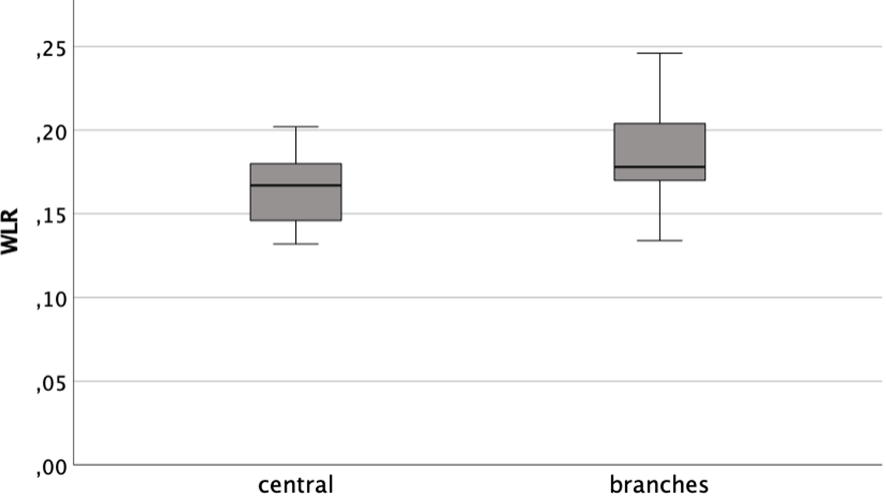
Box plot of WLR central arterioles and peripheral branches.

Distinguishing between the central arterioles and branches the mean WLR were 0.164 ( ± 0.019) and 0.185 (±0.026), respectively. The data was distributed normally (Kolmogorov-Smirnov-Test *p*_central_=0.2 and *p*_branch_=0.072). The arithmetic means differed significantly (t-test T=-6.180; *p*<0,001). However, they showed a positive correlation to each other (Pearson *r_P_*_=_0.494; *p*<0.001) (see **Figure 3**).

#### 3.1.1 WLR and age

There were no significant differences between the age groups for both the central arterioles and the branches (ANOVA F_central_(4,45)=0.560, p=0.693; F_branches_(4,45)=0.788, p=0.539) (see **Table 2**). There was also no significant correlation with age and the WLR at the central arterioles (Pearson *r_P_* =0.076; *p*=0.599) or branches (Pearson *r_P_* =-0.072; *p*=0.621).

**Table 2:** WLR, WCSA and LD for all vessels, branches and the central arterioles in all age groups.

age	arteries	n	mean	standard deviation	mean	standard deviation	mean	standard deviation
			WLR		WCSA		LD	
all ages	Arterioles	50	0.164	0.019	27673.130	495.073	99.207	7.235
	branches	50	0.185	0.026	1189.360	378.875	60.545	9.243
20-29	arterioles	10	0.163	0.016	2782.241	526.917	99.548	8.417
	branches	10	0.181	0.026	1219.040	264.593	62.604	6.056
30-39	arterioles	10	0.168	0.023	2968.247	518.282	102.234	4.791
	branches	10	0.198	0.033	1423.6252	452.903	´64.054	9.587
40-49	arterioles	10	0.157	0.018	2648.739	579.518	98.526	7.642
	branches	10	0.181	0.019	1068.003	413.918	57.303	10.130
50-59	arterioles	10	0.164	0.023	2698.625	506.628	98.470	8.081
	branches	10	0.181	0.019	1111.252	357.785	58.910	7.245
60-69	arterioles	10	0.169	0.017	2717.798	356.441	97.256	7.202
	branches	10	0.182	0.031	1124.881	342.551	59.856	12.160

#### 3.1.2 WLR and blood pressure

There was no relevant correlation of WLR of the central arterioles to SBP (Pearson *r_P_* =0.118 *p*=0.414) or DBP (Pearson *r_P_* =0.106 *p*=0.463) or MBP (Pearson *r_P_* =0.125 *p*=0.388).

**Figure 4:**
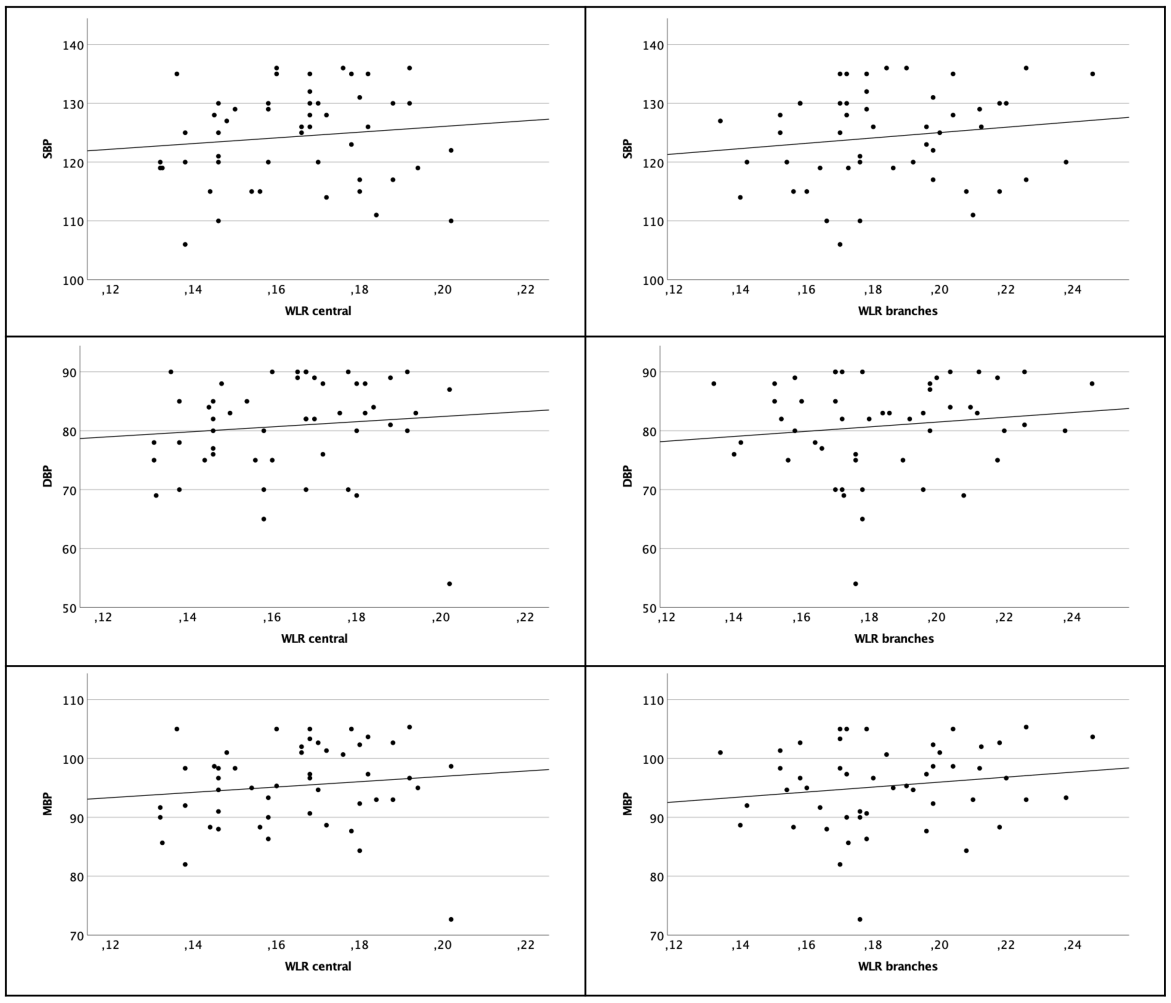
Scatter plot of WLR central arterioles (left column) and branches (right column) and SBP, DBP and MBP. There are no significant correlations.

Considering the more peripheral branches there was also no significant correlation to the blood pressure parameters [SBP (Pearson *r_P_* =0.149 *p*=0.300); DBP (Pearson *r_P_* =0.133 *p*=0.356) and MBP (Pearson *r_P_* =0.157 *p*=0.276)] (see **Figure 4**).

#### 3.1.3 WLR and gender

Comparing the mean of WLR between genders there was no significant difference (t-test T_central_=0.765 *p*_central_=0.448; t-test T_branches_=1.385 *p*_branches_=0.172).

#### 3.1.4 WLR and IOP

**Figure 5:**
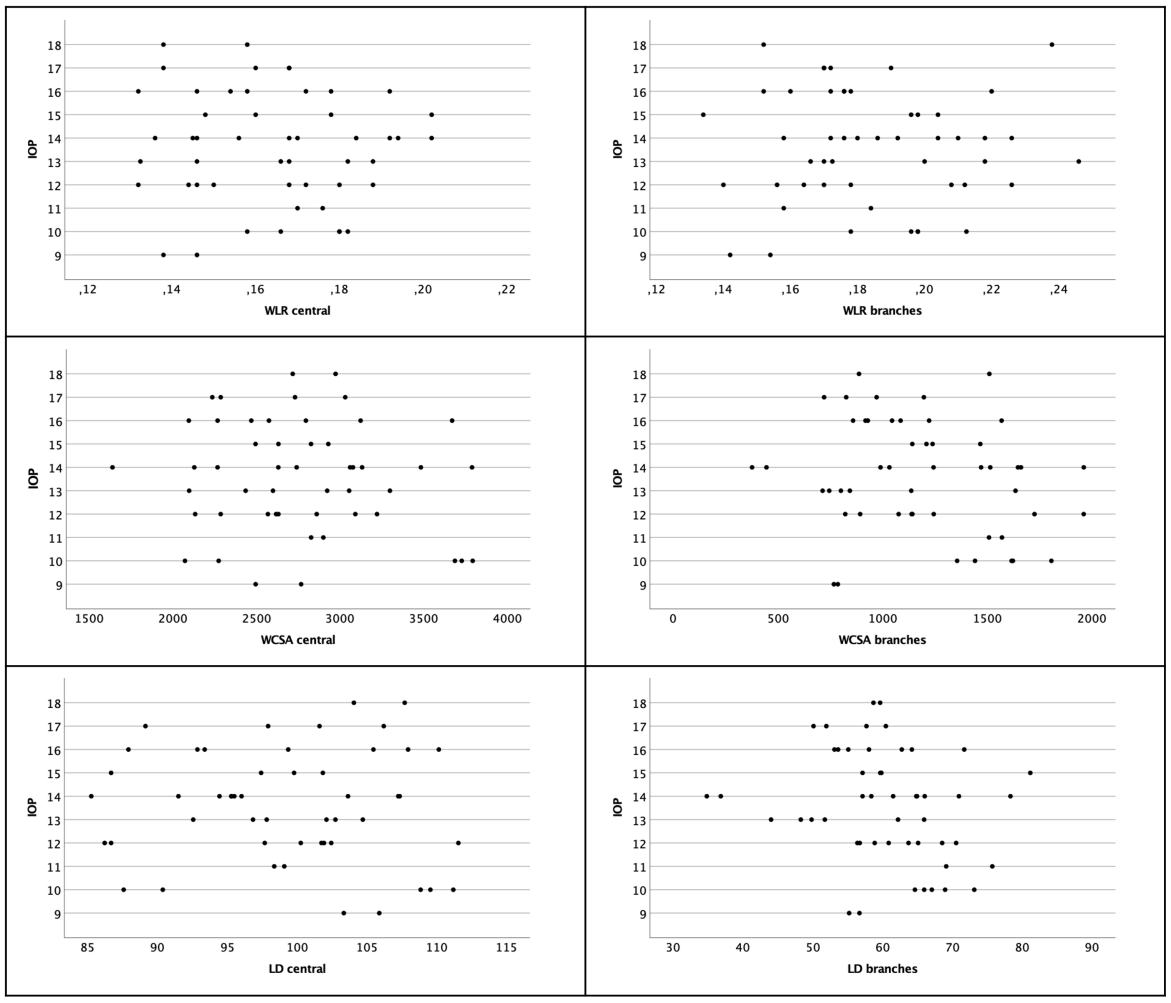
Scatter plot of WLR, WCSA and LD with regard to IOP. There was no significant correlation. Left column shows central arterioles, right column shows branches.

There was no association found for WLR and IOP on both locations (Pearson *r_central_* =-0.065; *p*=0.654; Pearson *r_branches_* =0.031; *p*=0.927) (see **Figure 5**).

### 3.2 WCSA

Mean WCSA at the central arterioles was 27673.130 (±495.073). For the branches, mean WCSA was 1189.360 (±378.875). There was a significant difference between values taken at the central arterioles and values at the branches for WCSA (T_WCSA_= 23.982 p<0,001). WCSA of central arterioles and peripheral branches correlated significantly positively (Pearson *r_P_* =0.462; *p*=0.001). WCSA showed a normal distribution on both locations (Kolmogorov-Smirnov-Test *p*_central_=0.2; *p*_branches_=0.2).

#### 3.2.1 WCSA and age

There were no significant differences between the age groups for both the central arterioles and the branches (ANOVA F_central_(4,45)=0.610, p=0.658; F_branches_(4,45)=1.459, p=0.231). There was also no significant correlation with age and the WCSA at the central arterioles (Pearson *r_P_* =-0.107; *p*=0.458) or branches (Pearson *r_P_* =-0.169; *p*=0.242).

#### 3.2.2 WCSA and blood pressure

Correlating WCSA of the central arterioles to SBP (Pearson *r_P_* =-0.021 *p*=0.932), to DBP (Pearson *r_P_* =-0.163 *p*=0.260) and MBP (Pearson *r_P_* =0.118 *p*=0.414) there was no relevant correlation.

Considering the more peripheral branches there was also no significant correlation to the blood pressure parameters [SBP (Pearson *r_P_* =0.112 *p*=0.438); DBP (Pearson *r_P_* =0.044 *p*=0.763) and MBP (Pearson *r_P_* =0.075 *p*=0.603)].

#### 3.2.3 WCSA and gender

Comparing the mean of WCSA of central arterioles and branches between genders there was no significant difference (t-test T_central_=-1.572 *p*_central_=0.123; T_branches_=2.030 *p*_branches_=0.05).

#### 3.2.4 WCSA and IOP

There was no association found for WCSA and IOP on both locations (Pearson *r_central_* =-0.124; *p*=0.389; Pearson *r_branches_* =-0.213 *p*=0.138) (see **Figure 5**).

### 3.3 LD

There was a significant difference between values taken at the central arterioles and values at the branches for LD (T_LD_=25.939 *p*<0,001). They did not show a relevant correlation (Pearson *r_P_*_=_0.200; *p*=0.164). Mean LD at the central arterioles was 99.207 (±7.235). For the branches, mean LD was 60.545 (±9.243). LD showed a normal distribution on both locations (Kolmogorov-Smirnov-Test *p*_central_=0.2; *p*_branches_=0.2).

#### 3.3.1 LD and age

There were no significant differences between the age groups for both the central arterioles and the branches (ANOVA F_central_(4,45)=0.654, p=0.627; F_branches_(4,45)=0.875, p=0.486). There was also no significant correlation with age and the LD at the central arterioles (Pearson *r_P_* =-0.183; *p*=0.202) or branches (Pearson *r_P_* =-0.142; *p*=0.326).

#### 3.3.2 LD and blood pressure

There was no relevant correlation of LD of the central arterioles to SBP (Pearson *r_P=_* 0.083 *p*=0.568) or DBP (Pearson *r_P_* =0.124 *p*=0.392) or MBP (Pearson *r_P_* =0.062 *p*=-0.668).

Considering the more peripheral branches there was also no significant correlation to the blood pressure parameters [SBP (Pearson *r_P_* =-0.078 *p*=0.591); DBP (Pearson *r_P_* =-0.024 *p*=0.869); MBP (Pearson *r_P_* =0.011 *p*=-0.938)].

#### 3.3.3 LD and gender

Gender had no influence on the LD of both the central arterioles or peripheral branches (t-test T_central_=1.691 *p*_central_=0.097; T_branches_=1.588 *p*_branches_=0.119)

#### 3.3.4 LD and IOP

The IOP and LD were not correlated (Pearson *r_central_* =-0.055; *p*=0.703; Pearson *r_branches_* =-0.219; *p*=0.126) (see **Figure 5**).

## 4 Discussion

Adaptive Optics (AO) imaging allows for the detailed examination of retinal vasculature at a near-microscopic level. Addressing a gap in available data, our study aimed to establish reference values for Wall-to-Lumen Ratio (WLR) across different age groups and at varying retinal locations, as outlined in **Table 2**. WLR serves as an important nondimensional parameter for indicating significant anatomic changes, such as stenosis.

In our investigation, the average WLR for central arterioles was 0.164 ( ± 0.019), and for arteriolar branches, it was 0.185 ( ± 0.026). An ANOVA analysis revealed no significant difference between the age groups. In this healthy, normotensive cohort no relevant correlation was found to blood pressure parameters.

The relationship between WLR and age has been explored in various studies. Meixner et al. found a highly significant correlation of WLR with age, noting a marked increase in older subjects compared to those under 40 years (10). Similarly, Streese et al. observed significant WLR differences in normotensive, non-smoking individuals of varying ages (16). Arichika et al. also identified a relevant correlation between WLR and age (17). In contrast, findings from Mehta et al., similar to our results, indicated no significant correlation of WLR with age (18).

In our study, only healthy subjects were included, and extreme values did not influence the results. All subjects had blood pressure below 140 mmHg. Our results align with findings from Koch et al., who reported that blood pressure had a more substantial effect on WLR than age. They investigated smaller vessels, which is consistent with our observations in smaller branches near the macula. Koch et al. included subjects with higher, unmedicated blood pressure. 19 of 49 subjects had SBP over 140 mmHg (9). Aging as suggesting our data might not play the major role. We controlled for the factor ‘increased untreated blood pressure’ by only including subjects with non-elevated blood pressure.

Retinal vascular diameter is a critical biomarker for assessing cerebrovascular and cardiovascular risks. Conditions like strokes, coronary heart disease, and cardiovascular mortality have been linked with narrowed arterioles and dilated venules. Streese et al. highlighted that retinal vascular diameter could improve cardiovascular risk assessments by 21% (16). A post-mortem study on stroke patients also demonstrated a strong correlation between cerebral and retinal vascular changes (18).

In this study, we compared smaller vessels with larger ones within the same individuals, primarily focusing on WLR as a cardiovascular risk parameter. Significant differences in WLR were found between smaller and larger vessels, indicating more pronounced parietal diameter and lumen narrowing in smaller arterioles, consistent with other studies that focused exclusively on smaller vessels (9).

Our findings underscore the potential of AO imaging in assessing systemic vascular conditions and retinal vessels. AO imaging is particularly useful in inherited retinal degenerative diseases (IRDs), where it can assess not only photoreceptor degeneration but also vascular changes. For IRDs, early vascular changes are crucial indicators, and our study provides a baseline for evaluating such changes in young patients with minimal vascular comorbidities.

Furthermore, our study suggests that AO imaging can monitor genetic treatments by assessing longitudinal vessel changes. In a recent study involving voretigene neparvovec, we observed stability in WLR, WCSA, and LD post-treatment over a 12-month period (15). The results presented here allow for the comparison of absolute values across different age groups and for an understanding the retinal effects in genetic therapies considering normative values.

However, it is important to note that the resolution in AO-imaging varies with location, which influenced our results. Challenges in obtaining good resolution near the optic disc due to factors like the curvature of the bulbus affected the accuracy of our measurements. When measuring the parameters with the given software, assessment is easier at bigger lumen sizes resulting in possible elevated diameters in smaller vessels.

Additionally, the study’s demographic was limited to Caucasians, and there’s a need to include more diverse ethnicities and older age groups in future research, especially given the increasing cardiovascular risk with age. We also did not consider the systolic to diastolic amplitude, a factor measured by other study groups (9), which may be an area for further exploration.

AO is capable of examining vascular alterations in arterioles at an almost microscopic level. While age did not seem to alter WLR, blood pressure alterations might do so. Therefore, AO-based vessel analysis may provide clinically useful biomarkers for cardiovascular health and should be tested in future studies.

## Data availability statement

The raw data supporting the conclusions of this article will be made available by the authors, without undue reservation. Please contact the corresponding author for the data sheet by e-mail.

## Ethics statement

The studies involving humans were approved by Ethics Commitee of the University of Tübingen. The studies were conducted in accordance with the local legislation and institutional requirements. The participants provided their written informed consent to participate in this study. Written informed consent was obtained from the individual(s) for the publication of any potentially identifiable images or data included in this article.

## Author contributions

FK: Conceptualization, Data curation, Formal analysis, Investigation, Methodology, Visualization, Writing – original draft. DM: Investigation, Writing – original draft. MR: Writing – review & editing. LK: Validation, Writing – review & editing. RJ: Validation, Writing – review & editing. SH: Investigation, Writing – review & editing. KrS: Project administration, Supervision, Writing – review & editing. KaS: Project administration, Resources, Supervision, Writing – review & editing. MK: Conceptualization, Data curation, Formal analysis, Investigation, Methodology, Visualization, Writing – original draft.
